# Comparison of four breast lymphoedema patient reported outcome measures in women following breast conserving surgery and radiotherapy: examination of frequencies of symptoms and measurement properties

**DOI:** 10.1007/s12282-026-01919-3

**Published:** 2026-10-08

**Authors:** Nicola Fearn, Sharon Kilbreath, Elizabeth Dylke, Wei Wang, Catalina Llanos, Verity Ahern, Elisabeth Elder, Farid Meybodi, Nicholas Olsen, Kirsty Stuart

**Affiliations:** 1 Sydney School of Health Sciences, Faculty of Medicine and Health, The University of Sydney, Sydney, NSW Australia; 2 Westmead Breast Cancer Institute, Westmead Hospital, Westmead, PO Box 143, Sydney, NSW Australia; 3 Sydney Medical School, The University of Sydney, Sydney, NSW Australia; 4 Sydney West Radiation Oncology Network, Westmead Hospital, Sydney, NSW Australia; 5 Stats Central, University of New South Wales, Sydney, NSW Australia

**Keywords:** Breast symptoms, Breast oedema, Clinimetrics, Measurement properties, Breast cancer, Radiotherapy

## Abstract

**Background:**

Lymphoedema of the breast is a potential consequence of breast cancer treatment. Questionnaires exist to quantify breast symptoms but evidence for measurement properties is limited. This study aimed to evaluate measurement properties of breast lymphoedema questionnaires and report on frequency and severity of breast symptoms in women with breast cancer.

**Methods:**

Women following breast conserving surgery and radiotherapy were recruited. Four questionnaires, Breast Lymphoedema Severity Symptom questionnaire (BLYSS), European Organisation for Research and Treatment of Cancer Quality of Life Questionnaire - Breast Symptom Subscale (EORTC-QLQ-BSS), Lymphoedema Symptom Intensity and Distress scale–Trunk (LSIDS-T) and Breast Oedema Bother scale (BOB), were completed three and nine-months after radiotherapy. Measurement properties and breast symptoms were evaluated.

**Results:**

Questionnaires were completed by 164 women at three-months, and 152 at nine-months post radiotherapy. Internal consistency was strong for all questionnaires (McDonald’s Omega and Cronbach’s ⍺ ≥0.7). Correlation between BLYSS and EORTC-QLQ-BSS and BOB visual analogue scale (BOB-VAS) met the hypothesised level (r_s_ ≥ 0.7) demonstrating construct validity. Exploratory factor analysis demonstrated single factors for individual tools and four factors when all four questionnaires were combined: pain, swelling, breast sensations and hardness. Responsiveness was high for BLYSS and moderate for EORTC-QLQ-BSS and BOB-VAS. Over half the women experienced symptoms ≥twice/week at mild-extreme severity. Breast pain, hardness, oversensitivity and swelling were most reported symptoms.

**Conclusion:**

The BLYSS demonstrated the strongest measurement properties among the PROMs evaluated with strong internal consistency, construct validity and responsiveness. Breast symptoms were commonly experienced by women after breast cancer treatment.

**Supplementary Information:**

The online version contains supplementary material available at https://doi.org/10.1007/s12282-026-01919-3.

## Introduction

Breast conserving surgery and radiotherapy (breast conservation treatment), whenever feasible, is the preferred treatment approach for local or locally advanced breast cancer due to improved survival [1–4], cosmesis and quality of life compared to mastectomy [5]. Lymphoedema of the breast (termed breast lymphedema in this manuscript), a chronic swelling of the breast region, is a possible side effect when a breast conservation approach is taken [6]. Reported incidence for breast lymphoedema varies, ranging from 1 to 90% depending on the measurement approach used to diagnose lymphoedema [6, 7]. Currently, no gold standard measurement approach for the diagnosis and assessment of breast lymphoedema exists [6, 8, 9]. In part, this is due to the complexity of breast lymphoedema.

Numerous factors contribute to the complexity of breast lymphoedema assessment. First, measurement of volume change caused by swelling is not accurate because surgery and radiotherapy may change the size of the breast. Second, skin thickness and tissue water may also be confounded by changes from radiotherapy [10, 11], oestrogen and progesterone fluctuations during the menstrual cycle [12–14], pregnancy and lactation [15], menopause and breast cancer endocrine treatment [12, 16–19]. Third, bioimpedance spectroscopy may not be accurate due to the breast shape and position on the chest wall [20]. Finally, breast lymphoedema can occur superficially, and/or deep in the parenchyma [21, 22]. However, common physical measures for breast lymphoedema, such as ultrasound and tissue dielectric constant, only measure superficial signs [23]. These factors all affect the utility of physical measures for breast lymphoedema diagnosis and monitoring.

Patient reported outcome measures (PROMs) may be used in screening for breast lymphoedema by providing insights into symptom severity that is not captured by physical measures. Numerous breast lymphoedema signs and symptoms are reported in the literature including swelling, volume changes, heaviness, hardness, aching, pain, pitting, peau d’orange, and skin redness [6, 7, 9, 24–26]. However, the symptom severity and frequency for women undergoing breast conserving treatment is currently unclear.

Several PROMs have been developed to quantify breast lymphoedema symptoms [24, 27–32]. However, as revealed by a recent systematic review [20], there is only limited evidence for measurement properties, particularly internal consistency and responsiveness, highlighting the need for more research. The systematic review and Consensus-based Standards for the selection of health Measurement INstruments (COSMIN) appraisal found no PROM could currently be recommended due to limited evidence for measurement properties [20].

The aims of this study were to (1) report on measurement properties for four breast lymphoedema PROMs (structural validity and internal consistency, construct validity and responsiveness) and (2) explore frequency and severity of breast symptoms reported by women following breast-conserving surgery and radiotherapy.

## Patients and methods

Women treated with breast conserving surgery and radiotherapy for local or locally advanced breast cancer at a single metropolitan cancer centre in Australia were recruited to a longitudinal study exploring breast lymphoedema incidence and risk factors (*manuscript submitted simultaneously for publication*). Women were recruited from a single metropolitan cancer centre in Australia at the time of consenting for, or during, radiotherapy, with measures captured between February 2020 and February 2022. Women completed four PROMs three- and nine-months after completing radiotherapy. This study was performed in line with the principles of the Declaration of Helsinki. Approval was granted by Western Sydney Local Health District Human Research Ethics Committee (2019/ETH12199) prior to commencing recruitment and all women provided written informed consent.

The results presented are a secondary analysis of data from the original incidence and risk factor study. The COSMIN study design checklist of PROMs [33] and reporting guideline for assessment and reporting of measurement properties were followed [34] (Online Resource 1).

### Participants

Women were eligible for enrolment if they were 18 years or older and had received breast-conserving surgery and radiotherapy, with or without chemotherapy. Women were excluded if they had received a mastectomy, breast expander or breast implants, previously had breast cancer surgery or radiotherapy to the chest or breast or were unable to provide informed consent in English.

### Data collection

Women were emailed a Qualtrics (www.qualtrics.com) link to complete questionnaires at three- and nine-months after completing radiotherapy. If a response was not received from the email invite, a telephone reminder was provided, where participants were offered to complete the questionnaires verbally (*n* = 4). Demographic questions were included in the online survey and information on cancer history and treatment, age, height, weight, menopausal status and medications was extracted from the medical notes.

### Included questionnaires

1. Breast Lymphoedema Symptoms Severity (BLYSS) [28]

The BLYSS questionnaire was developed to measure breast lymphoedema following breast cancer. Women with breast lymphoedema and clinicians were consulted to develop and validate the questionnaire items [28]. Only the first subscale was used in this study which includes seven items rated on a five-point Likert scale from *none (0)* to *extreme (4)*, total score range 0–28.

Published measurement properties include content validity (sufficient with moderate evidence), reliability (sufficient with very low evidence) and construct validity (indeterminate with very low evidence) [20].

(2) European Organization for Research and Treatment of Cancer Breast Cancer-Specific Quality of Life Questionnaire – Breast symptom subscale (EORTC-QLQ-BSS) [27]

The EORTC-QLQ-BSS is embedded in the quality-of-life tool for breast cancer (EORTC-QLQ-BR23). This symptom subscale was not developed for the purpose of breast lymphoedema diagnosis and has not been validated with women with breast lymphoedema. Four breast symptoms questions are rated on a four-point Likert scale ranging from *not at all* to *very much.* Published measurement properties for the EORTC-QLQ-BR23 include content validity (sufficient with low evidence), internal consistency (indeterminate with low evidence), reliability (indeterminate with very low evidence), construct validity (sufficient with low evidence) and responsiveness (sufficient with low evidence) [20].

(3) Lymphedema Symptom Intensity and Distress -Trunk (LSIDS-T) [32]

LSIDS-T was developed based on a literature search and expert opinion but did not involve women with breast lymphoedema during development. A pre-published version was used in this study, with permission from developers, and differs slightly to the published version [32]. Items that do not appear in the published version were removed from analysis in this study. The soft tissue subscale includes six symptoms related to breast lymphoedema and was analysed for this study. Respondents first answer *yes* or *no* to the presence of each symptom and then rate intensity and distress from *slight (1)* to *severe (5)*.

Published measurement properties include construct validity (sufficient with very low evidence), content validity and structural validity (indeterminate with very low evidence), and internal consistency (indeterminate with moderate evidence) [20].

(4) Breast Oedema Bother scale (BOB) *– (Refer to ESM 2 for details)*

The BOB questionnaire was developed by author KS and includes four questions relating to “breast bother”, a term encompassing breast lymphoedema symptoms including swelling, pain, discomfort, heaviness, tenderness, aching and/or hardness. Women rate frequency of breast bother (0 *- not at all*,* 1 - monthly*,* 2 - fortnightly to once a week*,* 3 - twice or more a week*, or *4 - everyday*). Average level and worst level of breast bother in the past week are then rated on a five-point scale (*not at all* to *extreme)*. The fourth question asks women to rate how much the breast bothered them in the past week on a 10 cm visual analogue scale (VAS).

This tool was developed using the clinical experience of radiation oncologists and breast surgeons. It has not undergone content validity testing with women with breast lymphoedema. It was developed to assist with rapid screening for breast concerns to allow triage of referrals to lymphoedema therapists.

### Data analysis

Statistical analysis was conducted using Statistical Package for the Social Sciences (SPSS, IBM, version 28.1).

Construct validity, internal consistency and structural validity analyses were completed using the three-month responses due to the larger sample size. Both timepoints were used for responsiveness (validity of the change score) analysis. Descriptive statistics were used for demographic data and questionnaire responses for each item at both timepoints.

Internal consistency was completed to determine how related the different items on each of the questionnaires were [35] and was analysed for each questionnaire using McDonald’s Omega and Cronbach’s alpha coefficient (⍺) (cut off values: ⍺ <0.5 unacceptable; 0.5–0.6 weak; 0.6–0.7 acceptable; 0.7–0.9 good; >0.9 excellent) [36, 37]. Missing data for EORTC-BR23, BLYSS and LSIDS soft tissue intensity items were handled using multiple imputation in IBM SPSS Statistics. Missingness on continuous and scale items was evaluated. To account for potential bias from missing values m = 5 imputed datasets were generated using the automatic method in SPSS.

Two hypotheses were tested to establish construct validity.

1. Strong positive (r_s_≥0.70) Spearman correlation coefficients expected between each questionnaire total score (BLYSS, EORTC-QLQ-BSS, LSIDS-T soft tissue subscale and BOB VAS).
2. Moderate to strong (r_s_ ≥0.50) Spearman correlation coefficients expected for women who identified breast swelling with total scores for the questionnaires, as above.

Structural validity was completed to determine the extent to which the four questionnaires reflect the dimensionality of breast lymphoedema [35]. Exploratory factor analysis (EFA) was chosen as no previous factor analysis has been conducted for breast lymphoedema questionnaires and due to the complexities of breast lymphoedema symptoms and association with treatment effects. Principal axis factoring was used to explore unidimensionality of the questionnaires measuring the construct of *breast lymphoedema*. EFA was conducted for each questionnaire individually, and then with all items from the four questionnaires combined. The cut-off for an absolute loading factor greater than 0.4 was selected for each factor [38]. Oblique rotation using direct Oblimin and Kaiser normalisation was completed as it was assumed all items are correlated [38]. There was no difference in factors when Orthogonal rotation was conducted (results not presented).

Hypothesis testing to establish responsiveness (validity of the change score) was conducted for the four questionnaires resulting in 33 hypotheses, as each tool was compared with each other (Table 4). Responsiveness was rated as high if 25% or less of the hypotheses were rejected, moderate if 25%-50% were rejected and poor if more than 50% were rejected [39].

1. Moderate positive (r_s_≥0.40) Spearman correlation coefficients expected between the total score change for the four measures: BLYSS questionnaire (H_0_ 1–3), EORTC-QLQ-BSS (H_0_ 1, 10, 11), LSIDS-T soft tissue subscale (H_0_ 2, 10, 18), and BOB VAS (H_0_ 3, 11, 18).
2. Moderate positive (r_s_≥0.40) Spearman correlation coefficients expected between the questionnaire totals/BOB VAS change and each BOB question 1–3 change (H_0_ 4–6, 12–14, 19–21, 25–27).
3. Moderate positive (r_s_≥0.40) Spearman correlation coefficients expected between the questionnaire total/BOB VAS and each of the breast swelling questions from BLYSS, LSIDS-T and EORTC-QLQ-BSS (H_0_ 7–9, 15–17, 22–24, 28–30).
4. Moderate-strong positive (r_s_≥0.50) Spearman correlation coefficients expected between the breast swelling questions from BLYSS, LSIDS-T and EORTC-QLQ-BSS (H_0_ 31–33).

### Sample size calculation

Sample size calculation was conducted for the purpose of identifying incidence and risk factors for the primary study (*n* = 160 required). However, a sample size of greater than 100 is generally suitable for the analysis of construct validity and responsiveness [35]. The sample size calculation for EFA is complex as EFA has not previously been performed for breast lymphoedema tools. However, a conservative sample size estimate for four factors is 170 participants, with a good level criterion (0.92) and high level of communality [40].

## Results

Two-hundred-and-two women consented to participate. Eight participants withdrew due to personal factors. One hundred and sixty-four women responded to the survey at three-months after radiotherapy. There appeared to be survey fatigue, with *n* = 164 completing the BOB scale (appeared first in the survey), *n* = 163 completed EORTC-QLQ-BSS (second), *n* = 161 completed LSIDS-T (third) and *n* = 158 completed BLYSS (last). Questionnaires were also completed at nine-months after radiotherapy, with a slightly lower response rate (*n* = 152 - BOB, EORTC-QLQ-BSS, LSIDS-T; *n* = 151 - BLYSS). The mean age of respondents was 58 years (SD 11.2years). Demographics are outlined in Table 1, with additional cohort details published elsewhere (*manuscript submitted simultaneously*).

**Table 1:** Demographics of participants

Demographics	Number (%) or Mean (SD)	
Age: Mean (SD) years	58.9 (11.2)	
Body mass index (BMI): Mean (SD) kg/m^2^	30.2 (6.5)	
< 25 kg/m^2^	37 (22.3%)	
25–29.99 kg/m^2^	53 (31.9%)	
≥ 30 kg/m^2^	76 (45.8%)	
Bra Cup size: n (%)		
A-B	69 (35.2%)	
C-D	75 (38.3%)	
DD-GG	52 (26.5%)	
Fitzpatrick skin type: n (%)		
1 (Pale white)	38 (19.4)	
2 (White)	65 (33.2)	
3 (Light brown)	57 (29.1)	
4 (Moderate brown)	24 (12.2)	
5 (Dark brown)	12 (6.1)	
6 (Deeply pigmented dark brown to black)	0 (0.0)	
Education Level: n (%)		
No school certificate/intermediate school certificate	37 (22.6%)	
High school certificate	29 (17.7%)	
Diploma/trade	38 (23.2%)	
University graduate	60 (36.6%)	
General Health (self-rated): n (%)		
Poor	7 (4.4%)	
Fair	25 (15.6%)	
Good	64 (40.0%)	
Very good	48 (30.0%)	
Excellent	16 (10.0%)	
Smoking: n (%)		
Never smoked	130 (67.0%)	
Previous smoker	51 (26.3%)	
Current smoker	12 (6.2%)	
Menopause status: n (%)		
Pre-menopause	29 (14.9%)	
Perimenopausal	22 (11.3%)	
Post-menopause	143 (73.7%)	
Marital status: n (%)		
Single	58 (29.9%)	
Married/De facto	136 (70.1%)	
Exercise: n (%)		
Less than 150 min per week	50 (31.3%)	
150 min or more per week	110 (68.8)	
Cancer stage: n (%)		
0-1B	125 (64.4%)	
2 A-3 C	69 (35.6%)	
Histologic grade: n (%)		
1	37 (19.0%)	
2	98 (50.0%)	
3	52 (26.7%)	
Tumour pathology		
ER positive: n (%)	154 (86.5%)	
PR positive: n (%)	133 (73.9%)	
HER2 positive: n (%)	18 (10.4%)	
Tumour size: Mean (SD) mm	25.2 (18.1)	
Surgery type: n (%)		
Wide local excision	161 (82.1%)	
Wide local excision + mastopexy/reduction	35 (17.9%)	
Re-excision required	51 (26.0%)	
Axillary surgery: n (%)		
Nil	26 (13.3%)	
Sentinel Node Biopsy (≤ 5 nodes)	143 (73.0%)	
Axillary lymph node dissection (> 5 nodes)	27 (13.8%)	
Number of lymph nodes removed: Mean (SD)	3.9 (± 5.8)	
Chemotherapy ± targeted therapy: n (%)		
Nil	118 (60.2%)	
Neo-adjuvant	37 (18.9%)	
Adjuvant	41 (20.9%)	
Endocrine treatment		
Yes	128 (65.3%)	
No	67 (34.2%)	
Missing	1 (0.5%)	
Radiotherapy regimen: n (%)		
Hypofractionated (40–42.5 Gy, 15–16#)	151 (77.0%)	
Conventionally fractionated (50 Gy, 25#)	45 (23.0%)	
Radiotherapy target volumes: n (%)		
Local (Breast only)	144 (73.5%)	
Locoregional (Breast and some/all lymph nodes)	52 (26.5%)	
Radiotherapy boost: n (%)	139 (70.9%)	

ER: oestrogen receptor, PR: progesterone receptor, HER2: hormone epidermal growth factor receptor 2, Gy: Gray, #: Fractions

### Measurement properties

#### Internal consistency (Table 2)

Cronbach’s alpha was excellent (BLYSS), or good (LSIDS -T soft tissue, BOB and EORTC-QLQ-BSS) with all questionnaires achieving ⍺≥0.7 (0.72 to 0.91). The five imputed Omega coefficients were compared against the baseline calculations. The imputed Omegas remained highly stable and tightly clustered around the baseline, confirming that filling the missing data did not introduce structural distortion or artificial variance.

**Table 2:** Internal consistency of four breast lymphoedema questionnaires

Breast questionnaire questions	McDonald’s Omega (ω) (95%CI)	Pooled imputed ω (range)	⍺ of total scale ⍺ if item removed	Median (IQR) [range]
BLYSS (*n* = 158)	0.91 (0.87–0.93)	0.90 (0.89–0.90)	0.91	
Q1 Swelling	0.89		0.89	1 (0–1) [0–4]
Q2 Pain	0.89		0.89	1 (0–1) [0–4]
Q3 Heaviness	0.89		0.89	0 (0–1) [0–3]
Q4 Unpleasant sensation	0.89		0.89	1 (0–1) [0–3]
Q5 Hardness	0.91		0.91	1 (0–1) [0–4]
Q6 Discomfort wearing a bra	0.89		0.89	0.5 (0–1) [0–4]
Q7 Indentations from the bra	0.89		0.89	0 (0–1) [0–4]
EORTC-BR23 Breast symptoms subscale (*n* = 163)	0.73 (0.65–0.80)	0.73 (0.72–0.74)	0.72	
Q50 Pain	0.67		0.59	2 (1–2) [1–4]
Q51 Swelling	0.70		0.67	1 (1–2) [1–4]
Q52 Oversensitive	0.64		0.61	2 (1–2) [1–4]
Q53 Skin problems	0.73		0.73	1 (1–2) [1–4]
LSIDS-T Intensity Soft tissue subscale (*n* = 161)	0.84 (0.77–0.89)	0.85 (0.84–0.86)	0.85	
Q1 Heaviness	0.		0.80	0 (0–1) [ 0–5]
Q2 Tightness	0.		0.81	0 (0–2) [0–5]
Q3 Pain	0.		0.82	0 (0–2) [0–5]
Q4 Achiness	0.		0.83	0 (0–1) [0–4]
Q5 Swelling	0.		0.83	1 (0–1) [0–4]
Q6 Hardness	0.		0.84	0 (0–2) [0–5]
BOB (*n* = 164)	0.91 (0.88–0.93)	No missing	0.83	
Q1 Frequency of breast bother	0.90		0.77	3 (1–4) [0–4]
Q2 Average breast bother in past week	0.88		0.79	1 (1–2) [0–3]
Q3 Worst breast bother in past week	0.87		0.78	1 (0–2) [0–4]
Q4 Breast bother VAS	0.88		0.87	2.6 (0.5-5) [0–10]

ω: McDonald’s Omega ⍺: Cronbach’s alpha (internal consistency), IQR: interquartile range, VAS: visual analogue scale, BLYSS: Breast Lymphoedema Symptoms Severity, EORTC-BR23: European Organization for Research and Treatment of Cancer Breast Cancer-Specific Quality of Life Questionnaire - Breast and Arm symptom sub-scales, LSIDS-T: Lymphedema Symptom Intensity and Distress -Trunk - Soft tissue subscale, BOB: Breast Oedema Bother scale

#### Construct validity (hypothesis testing) (Table 3)

The items from each questionnaire correlated with their total scores (r_s_=range 0.38–0.89). Correlations between the questionnaire total scores only achieved the hypothesised correlation strength (r_s_ ≥0.7) for the BLYSS with the BOB-VAS and EORTC-QLQ-BSS.

The BLYSS swelling item correlated strongly (r_s_=0.81) to the BLYSS total and moderately (r_s_=0.51–0.61) to the other three questionnaire totals, with all achieving the hypothesised correlation (r_s_≥0.50).

The multiple imputation analysis yielded results that were structurally identical to the original analysis, with no changes to the significance or direction of any hypothesized relationships. These findings confirm that the pattern of missing data did not introduce systematic bias or alter the overall construct validity conclusions of the scale.

**Table 3:** Construct validity of four breast lymphoedema questionnaires – hypothesis testing

Scale	No. of items	Mean	SD	Item-total correlation (*r*_s_)	Correlation with other tools (*r*_s_)			Correlation with BLYSS Breast swelling question (*r*_s_)
				Range	BLYSS	BOB VAS	LSIDS-T Soft tissue intensity	
BLYSS	7	5.4	4.9	0.60–0.80				0.80 (0.73–0.85)
EORTC-BR23	4	6.7	2.2	0.38–0.61	**0.71 (0.62–0.78)**	0.67 (0.57–0.75)	0.67 (0.62–0.75)	0.61 (0.51–0.70)
LSIDS-T Soft tissue intensity	8	4.7	5.8	0.43–0.73	**0.72 (0.64–0.79)**	0.64 (0.53–0.72)		0.61 (0.50–0.70)
BOB *BOB VAS*	4	7.7 3.1	5.4 2.7	0.72–0.89	**0.72 (0.63–0.78)**			0.51 (0.39–0.62)

Hypothesis 1: Strong correlation between each questionnaire total score for all four measures (r_s_≥0.70) (bold text indicates hypothesis conditions met)

Hypothesis 2: Moderate to strong correlation (r_s_≥0.50) for women who identified breast swelling on BLYSS with total scores on the BLYSS, LSIDS-T soft tissue subscale total scores and BOB VAS (bold text indicates hypothesis conditions met).

SD: standard deviation, *VAS: visual analogue scale, BLYSS: Breast Lymphoedema Symptoms Severity, EORTC-BR23: European Organization for Research and Treatment of Cancer Breast Cancer-Specific Quality of Life Questionnaire - Breast symptom sub-scale, LSIDS-T: Lymphedema Symptom Intensity and Distress -Trunk - Soft tissue subscale, BOB: Breast Oedema Bother scale, r_s_: Spearman correlation coefficient

#### Responsiveness (Table 4)

Hypotheses were tested for correlations between the total score change and change on individual items. BLYSS total score change correlated moderately with total score change on other questionnaires and individual items, except with the LSIDS-T swelling question (11% hypotheses rejected – high responsiveness). EORTC-QLQ-BSS correlated moderately with six of nine hypotheses, as did the BOB-VAS (33% hypotheses rejected – moderate responsiveness). LSIDS-T soft tissue change score correlated moderately for three of nine hypotheses (*n* = 6, 67% refuted- poor responsiveness).

**Table 4:** Responsiveness of four breast lymphoedema questionnaires change scores– hypothesis testing

	Hypothesis	Correlations *r*_s_	Confirmed		
Moderate positive (*r*_s_≥0.40) correlation expected between BLYSS questionnaire total change score:					
1	With EORTC Breast symptoms total	**0.45**	Yes		
2	With LSIDS-T soft tissue subscale total	**0.47**	Yes		
3	With BOB VAS	**0.47**	Yes		
4	With BOB Q1	**0.48**	Yes		
5	With BOB Q2	**0.46**	Yes		
6	With BOB Q3	**0.42**	Yes		
7	With BLYSS swelling	**0.62**	Yes		
8	With EORTC swelling	**0.47**	Yes		
9	With LSIDS-T swelling	0.35	No		
Moderate positive (r_s_≥0.40) correlation expected between EORTC breast symptoms total change score:					
10	With LSIDS-T soft tissue subscale total	0.37	No		
11	With BOB VAS	**0.43**	Yes		
12	With BOB Q1	**0.41**	Yes		
13	With BOB Q2	**0.44**	Yes		
14	With BOB Q3	**0.46**	Yes		
15	With BLYSS swelling	0.38	No		
16	With EORTC Swelling	**0.62**	Yes		
17	With LSIDS-T swelling	0.20	No		
Moderate positive correlation (r_s_≥0.40) expected between the LSIDS-T soft tissue subscale change score:					
18	with BOB VAS	**0.43**	Yes		
19	With BOB Q1	0.15	No		
20	WIth BOB Q2	0.20	No		
21	With BOB Q3	0.16	No		
22	With BLYSS swelling	0.39	No		
23	With EORTC swelling	0.34	No		
24	With LSIDS-T swelling	**0.54**	Yes		
Moderate positive (r_s_≥0.40) correlation expected between BOB VAS change score:					
25	With BOB Q1	**0.48**	Yes		
26	With BOB Q2	**0.70**	Yes		
27	With BOB Q3	**0.70**	Yes		
28	With BLYSS swelling	0.27	No		
29	With EORTC swelling	0.21	No		
30	With LSIDS-T swelling	0.21	No		
Moderate positive correlation (r_s_ ≥0.50) expected between the change score on swelling items:					
31	BLYSS swelling with EORTC swelling	**0.54**	Yes		
32	BLYSS Swelling with LSIDS-T swelling	0.44	No		
33	EORTC swelling with LSIDS-T Swelling	0.36	No		
Total amount of hypotheses that were rejected			14/33 (42%)		
BLYSS total hypotheses rejected			1/9 (11%)		
EORTC Breast symptoms total hypotheses rejected			3/9 (33%)		
LSIDS-T Soft tissue total hypotheses rejected			6/9 (67%)		
BOB VAS hypotheses rejected			3/9 (33%)		
Swelling questions hypotheses rejected			2/3 (66%)		

Bold denotes when correlation r_s_ value was above the 0.4 threshold and hypothesis confirmed

BLYSS: Breast Lymphoedema Symptoms Severity, EORTC-BR23: European Organization for Research and Treatment of Cancer Breast Cancer-Specific Quality of Life Questionnaire - Breast symptom sub-scale, LSIDS-T: Lymphedema Symptom Intensity and Distress -Trunk - Soft tissue subscale, BOB: Breast Oedema Bother scale, r_s_: Spearman correlation coefficient

Only the breast swelling questions on BLYSS and EORTC-QLQ-BSS correlated moderately with each other (r_s_ ≥0.50) with neither tool correlating to the hypothesised level with LSIDS-T breast swelling item.

However, as no objective measurement tool was used to determine clinical change in breast swelling, it is unclear how subjective changes are associated with objective changes.Structural validity (Table 5):

**Table 5:** Factor analysis: principal axis factoring using Oblimin rotation with Kaiser normalisation, Eigenvalues > 1

Breast questionnaire items	Factor 1 Pain	Factor 2 Swelling	Factor 3 Sensations	Factor 4 Hardness
BLYSS Q1 swelling		**0.817**		
BLYSS pain	**0.523**	0.392*		
BLYSS heavy		**0.762**		
BLYSS unpleasant sensation		**0.566**		
BLYSS hardness		0.320*		**0.465**
BLYSS bra discomfort		**0.689**		
BLYSS bra indentations		**0.782**		
EORTC BR23 pain	**0.672**			
EORTC BR23 swelling		**0.727**		
EORTC BR23 oversensitivity	0.381*			
EORTC BR23 skin changes				
BOB frequency of breast bother	**0.762**			
BOB average level of breast bother	**0.895**			
BOB worst level of breast bother	**0.917**			
BOB VAS breast bother	**0.757**			
LSIDS-T heaviness			**0.744**	
LSIDS-T tightness			**0.911**	
LSIDS-T pain			**0.550**	
LSIDS-T achiness			**0.499**	
LSIDS-T swelling		**0.636**		0.348*
LSIDS-T hardness				**0.779**

Bold denotes main factor

*Did not reach ≥0.4 loading cut-off

BLYSS: Breast Lymphoedema Symptoms Severity, EORTC-BR23: European Organization for Research and Treatment of Cancer Breast Cancer-Specific Quality of Life Questionnaire - Breast symptom sub-scale, LSIDS-T: Lymphedema Symptom Intensity and Distress -Trunk - Soft tissue subscale, BOB: Breast Oedema Bother scale

EFA of each individual questionnaire demonstrated that items loaded to a single factor (all loadings ≥ 0.4). An additional EFA combining all items from the four questionnaires revealed four factors (Table 5). Applying Kaiser’s criterion of eigenvalues greater than 1.0, four distinct factors were extracted, which combined to explain 70.10% of the total variance. Factor 1, pain, emerged as a dominant factor accounting for half of the total variance. BOB items loaded unidimensionally to the *pain* factor, along with questions related to *pain* and *oversensitivity* from BLYSS and EORTC-QLQ-BSS. A third factor was linked with items related to *breast sensations* from the LSIDS-T questionnaire including breast heaviness, tightness, pain and achiness. Items associated with the construct *breast swelling* included five of seven questions from BLYSS (breast swelling, heaviness, unpleasant sensation, bra discomfort and bra indentations) and swelling questions from EORTC-QLQ-BSS and LSIDS-T. *Breast hardness* was identified as a fourth factor with the breast hardness items from the BLYSS and LSIDS-T loading to this factor. The EORTC-QLQ-BSS skin changes item did not load to any of the four factors.

To account for missing data, Multiple Imputation was performed, yielding five imputed datasets. A Principal Axis Factoring with Direct Oblimin rotation was executed on each imputed matrix. The factor structures remained highly consistent across all five imputations, reliably extracting the dominant ‘pain factor’ and all items remaining loaded to the same factor as the original analysis.

### Frequency and severity of symptoms

BLYSS: Nearly 90% of women reported at least one breast symptom on the BLYSS (mild to extreme) at three-months after radiotherapy. Breast pain was most frequently reported (68%), followed by breast hardness (63%), unpleasant sensation (57%) and swelling (53%). Discomfort wearing a bra (50%), bra indentations (44%) and heaviness (44%) were reported less frequently.

At nine-months, breast pain (65%), hardness (60%) and unpleasant sensations (55%) continued to be the most frequently reported symptoms. Discomfort wearing a bra (54%), bra indentations (48%) and heaviness (46%) were more commonly reported at nine-months, despite the number of women reporting breast swelling reducing slightly (50%). Mild and moderate ratings were most commonly reported at both time points, but the proportion of women reporting higher levels (severe and extreme) increased at nine-months, especially for swelling (3.8% − 8.6%).

EORTC-QLQ-BSS: Breast pain was the most reported symptom at three-months, with over 70% of women reporting *a little bit* (52%), *quite a bit* (17%) or *a lot* (2.5%). Oversensitivity (55%) and swelling (45%) were also commonly reported with most women rating as *a little bit*.

At nine-months after radiotherapy, symptom prevalence reduced slightly for pain (59%), swelling (42%) and oversensitivity (54%).

LSIDS-T: Symptoms were less frequently reported on the LSIDS-T compared to the other questionnaires despite similarities in symptom terms; however, the intensity ratings were more evenly spread across the 1–5 rating options. Distress related to symptoms was higher at three-months post-radiotherapy compared to nine-months.

BOB: Half (51%) the women experienced breast symptoms twice or more a week (25%) or every day (26%) three-months after radiotherapy. Over 75% reported mild to extreme breast bother in the past week. The BOB-VAS mean was 3.1 (SD = 2.7, range 0–10). At nine-months after radiotherapy, 50% of women continued to report breast symptoms twice or more a week (23%), or everyday (27%) with 65% reporting mild to extreme breast bother in the past week. The VAS mean was lower at nine-months at 2.6 (SD = 2.6, range 0–10).

## Discussion

This study evaluated the measurement properties for three existing breast symptom questionnaires and one newly developed tool. Internal consistency was good to excellent for all four questionnaires, meeting the COSMIN guidelines for good measurement properties [35]. Construct validity was strongest for the BLYSS revealing that only the BLYSS correlated strongly with the EORTC-QLQ-BSS and BOB VAS, while the LSIDS-T soft tissue subscale did not achieve the hypothesised correlation strength with any other tool. Responsiveness was also highest for BLYSS and poorest for LSIDS-T. Breast symptoms were frequently reported, especially pain, at three and nine-months post-radiotherapy.

Factor analysis revealed some symptoms commonly included on breast lymphoedema questionnaires may load to broader factors other than *breast swelling*. Symptoms including pain, hardness and breast sensitivity loaded to separate factors. Breast pain was the most frequently reported symptom on the BLYSS and EORTC-QLQ-BSS, and the factor relating to pain emerged as the dominant factor, accounting for half of the total variance across the questionnaire items. When breast symptoms were grouped into a single term “breast bother” on the BOB, these loaded to the *pain* factor. Women can experience upper body and breast sensory symptoms due to lymphoedema, for example pain, tightness and numbness [41–43]. However, pain in the chest area is also common due to nerve and musculoskeletal issues caused by breast surgery or radiotherapy [44] and cording [45]. Breast sensitivity and pain may also be higher in women of younger age [44] and/or with smaller breasts [46]. In our study, the participants’ mean age was 58 years, which is lower than the mean age for breast cancer (62 years), however, breast cancer is the most commonly diagnosed cancer for 20–59 year olds [47]. 35% of women had a A-B bra cup size, which may also explain the high frequency of pain in our sample. Use of PROMs, whilst able to capture symptoms of the breast are not able to definitively identify breast lymphoedema as some symptoms may be treatment-related rather than due to lymphoedema. In the future, confirmatory factor analysis, building from these findings, should be done to further support the relationships between symptoms and individual factors.

The lack of a clear definition of breast lymphoedema hinders physical assessment and development of questionnaires. The tissue changes that can occur because of lymphoedema, such as increased dermal thickness, fibrosis and oedema, are also known tissue changes caused by radiotherapy [48] and can be present prior to radiotherapy as a result of surgery [49]. Additionally, it has been shown that physical measures used in breast lymphoedema do not directly correlate with patient symptoms [10]. Unique breast lymphoedema symptoms may exist, such as peau d’orange, but other signs such as skin redness, pain and discomfort, are also known post-treatment toxicities [50]. To improve breast lymphoedema diagnosis and measurement, a greater understanding of breast lymphoedema signs and symptoms is required.

The BLYSS, LSIDS-T and EORTC-QLQ-BSS, were the only tools, in English, developed to measure breast symptoms following breast cancer at the commencement of this study. Since that time, other valid and reliable tools have been published including the Breast Edema Questionnaire (BrEQ) in Dutch. This tool has not yet undergone cross-cultural validity testing for the English translation [24]. The items from the first subscale of the BrEQ are similar to the questions in the BLYSS with the addition of “skin redness”, “tensed skin” and “enlargement of the pores” in the BrEQ. Factor analysis for this tool has not yet been published, however, there is evidence for internal consistency, reliability and construct validity in a small (*n* = 55) sample and it has an established cut off for breast lymphoedema diagnosis/identification [24]. Another promising tool recently developed and tested in a small sample, the Breast Lymphoedema Symptom Questionnaire (BLSQ) has a diagnostic cut-off [51]. Both questionnaires used ultrasound as the reference criterion for breast lymphoedema diagnosis; however, ultrasound only measures the presence of superficial lymphoedema signs. Whilst these cutoffs may serve to identify superficial lymphoedema, they may not detect lymphoedema deeper in the breast [21, 22].

The EORTC-QLQ-BR23 Breast Symptom Subscale has been used in studies exploring breast oedema following breast cancer and is often used as a reference for the development of new breast lymphoedema tools [10, 29–31, 41, 51–56]. The EORTC-QLQ-BR23 breast symptom questions were developed with a general breast cancer population, not a specific sample of women following breast conserving surgery, nor with breast lymphoedema. Our study revealed that the BLYSS had superior measurement properties to the EORTC-QLQ-BSS. As more robust breast lymphoedema symptom scales now exist and should be used in preference of the EORTC-QLQ-BSS in future breast lymphoedema research and clinical practice. The EORTC-QLQ-BSS can, however, provide relevant breast lymphoedema screening information for clinicians who routinely deliver the EORTC-QLQ-BR23 questionnaire, as it correlated strongly with the BLYSS.

Breast symptoms were frequently reported in this study with little reduction over the two questionnaire timepoints. A recent systematic review exploring the relationship between upper-body symptoms and impairments following breast cancer treatment found a negative impact on quality of life [42] leading to disability [57]. Routine referral to cancer rehabilitation services is frequently not standard practice despite evidence in support of addressing the myriad of long-term effects of cancer treatment [57]. The high prevalence of breast symptoms highlights the need to routinely screen for, and address, these issues to improve quality of life.

Despite the strengths in this study, there were some limitations. The main limitation was that women did not undergo physical assessment for breast lymphoedema to allow correlation of physical breast changes with the symptoms reported. The measurement properties of the included tools, therefore, are relative to each other, rather than compared to objectively measured breast oedema presence. This study was conducted during the COVID-19 pandemic and all study activities were completed virtually following initial participant recruitment. Furthermore, physical assessment of breast lymphoedema is challenging with existing tools, other than ultrasound, having insufficient measurement properties for breast lymphoedema measurement, and no gold standard assessment [58], to allow comparison to the findings from PROMs. The design of the study also meant we could not explore reliability or measurement error and minimal clinically important differences of tools, all of which are measurement properties requiring further evidence for breast lymphoedema assessment. This study also only measured symptoms three- and nine-months after radiotherapy for breast cancer. Future studies are needed to measure how breast symptoms evolve over longer periods of time. Finally, all participants spoke English, impacting on the generalisability of the findings. At the time of the study, only the EORTC-QLQ-BSS had cross-cultural validation and translated versions of the assessment tool. Future research to translate and complete cross-cultural validation for breast lymphoedema PROMs is vital to ensure the impact of breast lymphoedema in diverse populations is captured.

The findings from this study provide additional evidence on the measurement properties of four breast lymphoedema symptom questionnaires. The BLYSS demonstrated the strongest measurement properties among the four PROMs evaluated. Additionally, this study demonstrates the range of breast symptoms are commonly experienced by women following breast conserving surgery and radiotherapy up to nine-months after completing radiotherapy.

## Supplementary Information

Below is the link to the electronic supplementary material.

Supplementary Material 1

Supplementary Material 2

## Data Availability

The datasets generated during and/or analysed during the current study are not publicly available as ethical approval for this was not sought but are available from the corresponding author on reasonable request.
