# Supplementary material for: Comparison of four breast lymphoedema patient reported outcome measures in women following breast conserving surgery and radiotherapy: examination of frequencies of symptoms and measurement properties
Source: Breast Cancer. 2026 Oct 8;33(6):1406–18. doi: 10.1007/s12282-026-01919-3 (PMC13695621; doi:10.1007/s12282-026-01919-3)
Supplement: Supplementary file 1 [file 12282_2026_1919_MOESM1_ESM.pdf]

COSMIN reporting guideline (Gagnier, J.J et al 2021)

General reporting recommendations relevant for all studies on measurement properties			
**Item number**	**Item name**	**Item description**	
Report section: title			
T1	Patient-reported outcome measure (PROM)	The name of the PROM instrument(s) (and version if relevant) being studied	N – specified four questionnaires
T2	Measurement property (MP)	What MPs are being studied or more generally, that MPs are being studied (if there are many properties being investigated, for example)	Y
T3	Study sample	General description of relevant study sample characteristics (e.g., condition of interest, language) and also any intervention or exposure (e.g., treatments) if applicable	Y
Report section: abstract			
A1	PROM	The name of the PROM instrument(s) (and version if relevant) being studied (i.e., the SF-36 or SF-12; language version) or if it concerns an item bank (e.g., PROMIS instruments). The type of instrument (e.g., a self-reported questionnaire or interview)	Y
A2	Measurement property	What MPs are being studied or more generally, that MPs are being studied (if there are many properties being investigated, for example)	Y
A3	Design	The type of study being used to test the properties (e.g., test–retest design, longitudinal study, cohort, cross sectional, case series, randomized etc.). Other details of the study design if relevant (intervention/exposure, description of comparison instruments, outcomes other than PROMs)	Y
A4	Sample	Inclusion / exclusion criteria. General description of relevant study sample characteristics (e.g., condition of interest, geographic location, language, other relevant demographic and baseline characteristics)	Y
A5	Methods	A brief description of the methods for investigating each MP including statistical analyses	P
A6	Results	The main results for all MPs investigated reporting statistics for each result with measures of precision where appropriate	P
A7	Discussion/ Conclusions	A brief description of the results in the context of existing evidence, main strengths and drawbacks and the need for future research on the PROM(s) investigated	N – insufficient word count
Report section: introduction			
I1	Name and describe the PROM of interest	Specify the name, type, language, and version of the PROM being investigated and how it was developed. Describe the construct the PROM aims to measure and its subscales; describe the structure of the PROM (e.g., the number of factors, the number of items, scoring algorithm); describe relevant instructions (like time period), and number or type of response categories. State whether the PROM is based on a reflective or formative model.<br>Note: This information may also appear in the methods section in greater detail	In methods

Comparison of four breast lymphoedema patient reported outcome measures in women following breast conserving surgery and radiotherapy: examination of frequencies of symptoms and measurement properties - Breast Cancer

Nicola Fearn, Sharon Kilbreath, Elizabeth Dylke, Wei Wang, Catalina Llanos, Verity Ahern, Elisabeth Elder, Farid Meybodi, Nicholas Olsen and Kirsty Stuart
Corresponding Author: Kirsty.stuart@health.nsw.gov.au

COSMIN reporting guideline (Gagnier, J.J et al 2021)

General reporting recommendations relevant for all studies on measurement properties			
Item number	Item name	Item description	
I2	Target population	Describe the specific target population that the PROM was designed for. The authors need to provide the appropriate and necessary characteristics of this population	Y
I3	Citation for the original development of the PROM	The citation for the original development paper(s) should be provided and other highly relevant citations related to the quality of the specific PROM under investigation	Y
I4	State of knowledge & Rationale	A description of the current scientific knowledge (what is known) regarding the MPs of? the PROM under investigation. The authors should provide a literature review or refer to a recent review of all existing evidence of the specific version (e.g., language, short form) of the PROM and explain why the new study is necessary and important. The rational for the current proposed study should be given	Y
I5	Definitions	Specialized terms should be defined or explained	N/A
I6	Objectives and hypotheses	State the specific objective(s) of the research and hypotheses related to the specific PROM under investigation	Y
Report section: general methods			
GM1	Study design	State the key elements of the study design	Y
GM2	Participants	State how the participants were chosen; the inclusion and exclusion criteria. (e.g., if a PROM for a specific condition, then the eligibility and selection criteria should reflect this)	Y
GM3	PROM administration	An explicit description of how and when the PROM(s) were administered (e.g., in what setting) including data collection devices/system used (e.g., paper-based, electronic administration / ePRO) should be provided	Y
GM4	Data collection procedures	Provide information about other data collection, exposure methods (e.g., allocation to interventions) and time points / follow-up points	Y
GM5	Power/sample size calculation	Provide a power calculation for all MP analyses. Alternatively, if a rule of thumb is used, state it and the source/citation	Y
GM6	Statistical analyses	Statistical analyses and tests corresponding to all hypotheses or objectives for all MPs should be reported. Where appropriate, a cutoff for statistical significance should be reported (e.g., p-value less than 0.05). A description of all statistics to be used to estimate the magnitude and direction of effect should also be reported, together with measures of variability or precision. Report statistical package used	Y
GM7	Missing data	State approaches or plan for dealing with missing data	N/A
GM8	Post hoc analysis	The report should specify analyses that used data after the data collection period concluded (i.e., if the analyses were post hoc; secondary data analyses) and describe the rationale for any post hoc analyses	Y
Report section: general results			

Nicola Fearn, Sharon Kilbreath, Elizabeth Dylke, Wei Wang, Catalina Llanos, Verity Ahern, Elisabeth Elder, Farid Meybodi, Nicholas Olsen and Kirsty Stuart
Corresponding Author: Kirsty.stuart@health.nsw.gov.au

COSMIN reporting guideline (Gagnier, J.J et al 2021)

General reporting recommendations relevant for all studies on measurement properties			
Item number	Item name	Item description	
GR1	Missing data	The amount and reasons for missing data should be explained for all analyses for all PROMs (or other outcome measurement instruments) and relevant groups	Y
GR2	Participant Characteristics	The study patients' characteristics should be described, including baseline PROM scores	Y
GR3	Sample size	If one study contained analyses using different sample sizes, the authors should report the sample size for each analysis	Y
Report section: discussion			
D1	MP evidence	Per measurement property the authors should compare the result to the criteria for good measurement properties (e.g., COSMIN criteria) [25], and determine if the specific MP is sufficient or not. Note: This information may also appear in the results section in greater detail in a table for example	Y Included in table
D2	Practical relevance	The authors need to discuss the practical relevance of the findings	Y
D3	Strengths and limitations	Strengths and limitations of the study should be discussed. For example, discuss if there were any significant potential biases in the study that could have impacted the results	Y
D4	Generalizability	Generalizability issues related to the PROM results should be discussed. For example, discuss if the results could be generalized to other populations given the sample studied	P
D5	Instrument changes	Discuss the need for modifications to the existing PROM or new PROM development. If you conclude that one of the measurement properties is insufficient, you could suggest some modification, or if it is really poor, you could suggest stopping use of the PROM (in the specific population or in general)	Y
D6	Future research	Report specifically the type of research needed to answer new questions arising out of these findings for the particular MP and PROM investigated	Y
Report section: conclusions			
C1	Conclusions	State the overall conclusions for each MP and of the use PROM investigated	Y
Report section: other information			
O1	Conflict of interest	State any relevant conflict of interest related to the PROM under investigation (e.g., an author being the PROM developer, funding body etc.)	Y
Specific reporting recommendations for studies on structural validity			
Item number	Item name	Item description	
SV1	Factor analyses: classical test theory (CTT) PROMs	Report details of the methods and results for any exploratory or confirmatory factor analyses. State the rational for any explorative factor analyses (e.g., no clear a priori hypotheses). For CFA, describe and justify the factor structure of tested models. Methods and results for checking of the assumptions should be described, the method	Y

COSMIN reporting guideline (Gagnier, J.J et al 2021)

General reporting recommendations relevant for all studies on measurement properties			
Item number	Item name	Item description	
		of estimation, goodness-of-fit statistics and cutoff points for good model fit, including factor loadings of best-fitting model	
SV2	Item Response theory (IRT) analyses	Type of IRT/Rasch model should be reported. Also report the method of estimation, methods and results for checking of the assumptions (unidimensionality (see factor analysis), local dependency (e.g., residual correlations), monotonicity; (e.g., Mokken scaling), goodness-of-fit statistics, and cutoff points for goodness of item/model fit, and all item parameters	N/A
Specific reporting recommendations for studies on internal consistency			
Item number	Item name	Item description	
IC1	Unit of measurement	Report internal consistency methods and results for each unidimensional scale or subscale. Report all evidence or assumptions associated with unidimensionality	Y
IC2	Continuous scores	Report Cronbach's alpha or omega statistics. Report other statistics calculated for internal consistency of continuous scores	Y
IC3	Dichotomous scores	Report Cronbach's alpha or Kuder–Richardson coefficient. Report other statistics calculated for internal consistency of dichotomous scores	NA
Specific reporting recommendations for studies on hypotheses testing for construct validity			
ConV1	Comparator instrument(s)	The comparator instruments should be appropriately described in terms of the construct(s) they intend to measure. Report the measurement properties of the comparator instruments and related citations or data	Y
ConV2	Comparator Group(s)	Report characteristics of groups being compared. Include sample sizes in each group	N/A
ConV3	Hypotheses	Report all hypotheses including the direction and magnitude of the expected correlations between the PROM of interest and another measurement instrument, or the direction and magnitude of differences in scores of the PROM between groups	Y
ConV4	Statistical analyses	Report all statistical methods and results used to test each hypothesis	Y
ConV5	Results	Report which specific results are in accordance with its hypothesis	Y

Code	
Y	Yes
N	No
P	Partially met
N/A	Not applicable

Comparison of four breast lymphoedema patient reported outcome measures in women following breast conserving surgery and radiotherapy: examination of frequencies of symptoms and measurement properties - Breast Cancer

Nicola Fearn, Sharon Kilbreath, Elizabeth Dylke, Wei Wang, Catalina Llanos, Verity Ahern, Elisabeth Elder, Farid Meybodi, Nicholas Olsen and Kirsty Stuart
Corresponding Author: Kirsty.stuart@health.nsw.gov.au
