# Supplementary material for: Comparison of four breast lymphoedema patient reported outcome measures in women following breast conserving surgery and radiotherapy: examination of frequencies of symptoms and measurement properties
Source: Breast Cancer. 2026 Oct 8;33(6):1406–18. doi: 10.1007/s12282-026-01919-3 (PMC13695621; doi:10.1007/s12282-026-01919-3)
Supplement: Supplementary file 2 [file 12282_2026_1919_MOESM2_ESM.pdf]

Electronic Supplementary Materials 2

BOB Scale - Breast Oedema Bother Scale

Women who have had breast conserving surgery and radiotherapy sometimes report symptoms of breast swelling, pain, discomfort, tenderness, heaviness, aching and/or hardness. These symptoms have been grouped into one term – **Bother.** Keeping these symptoms in mind, please circle the level or frequency that you experience any of these symptoms (breast bother).

1. How often does your breast bother you?				
Not at all	Not every month, up to monthly	Fortnightly to once a week	Twice or more a week	Every day
2. On average, what level has this breast bother been in the last week?				
Not at all	Mild	Moderate	Severe	Extreme
3. What has been the worst level of breast bother in the last week?				
Not at all	Mild	Moderate	Severe	Extreme
4. Overall, how much has your breast bothered you in the last week (mark a point on the line)?				
Not at all			Extreme	

Comparison of four breast lymphoedema patient reported outcome measures in women following breast conserving surgery and radiotherapy: examination of frequencies of symptoms and measurement properties - Breast Cancer

Nicola Fearn, Sharon Kilbreath, Elizabeth Dylke, Wei Wang, Catalina Llanos, Verity Ahern, Elisabeth Elder, Farid Meybodi, Nicholas Olsen and Kirsty Stuart

Corresponding Author: Kirsty.stuart@health.nsw.gov.au
